# Comparing anatomy, chemical composition, and water permeability of suberized organs in five plant species: wax makes the difference

**DOI:** 10.1007/s00425-022-03975-3

**Published:** 2022-08-21

**Authors:** Kiran Suresh, Viktoria V. Zeisler-Diehl, Tobias Wojciechowski, Lukas Schreiber

**Affiliations:** 1 Institute of Cellular and Molecular Botany, Department of Ecophysiology, University of Bonn, Kirschallee 1, 53115 Bonn, Germany; 2 IBG-2, Forschungszentrum Jülich, Wilhelm-Johnen-Straße, 52428 Jülich, Germany

**Keywords:** Bark, Diffusion barrier, Periderm, Suberization, Storage root, Transpiration, Tuber, Water loss, Wax

## Abstract

**Main conclusion:**

The efficiency of suberized plant/environment interfaces as transpiration barriers is not established by the suberin polymer but by the wax molecules sorbed to the suberin polymer.

**Abstract:**

Suberized cell walls formed as barriers at the plant/soil or plant/atmosphere interface in various plant organs (soil-grown roots, aerial roots, tubers, and bark) were enzymatically isolated from five different plant species (*Clivia miniata*, *Monstera deliciosa*, *Solanum tuberosum*, *Manihot esculenta*, and *Malus domestica*). Anatomy, chemical composition and efficiency as transpiration barriers (water loss in m s^−1^) of the different suberized cell wall samples were quantified. Results clearly indicated that there was no correlation between barrier properties of the suberized interfaces and the number of suberized cell layers, the amount of soluble wax and the amounts of suberin. Suberized interfaces of *C. miniata* roots, *M. esculenta* roots, and *M. domestica* bark periderms formed poor or hardly any transpiration barrier. Permeances varying between 1.1 and 5.1 × 10^−8^ m s^−1^ were very close to the permeance of water (7.4 × 10^−8^ m s^−1^) evaporating from a water/atmosphere interface. Suberized interfaces of aerial roots of *M. deliciosa* and tubers of *S. tuberosum* formed reasonable transpiration barriers with permeances varying between 7.4 × 10^−10^ and 4.2 × 10^−9^ m s^−1^, which were similar to the upper range of permeances measured with isolated cuticles (about 10^−9^ m s^−1^). Upon wax extraction, permeances of *M. deliciosa* and *S. tuberosum* increased nearly tenfold, which proves the importance of wax establishing a transpiration barrier. Finally, highly opposite results obtained with *M. esculenta* and *S. tuberosum* periderms are discussed in relation to their agronomical importance for postharvest losses and tuber storage.

## Introduction

Plant environment interfaces are formed by hydrophobized cell walls which are additionally modified by cutin and suberin polymers (Pollard et al. 2008). Outer epidermal cell walls of leaves facing the atmosphere are modified by the deposition of the plant cuticle (Riederer and Müller 2006). It is highly impermeable for water and it protects leaves from uncontrolled water loss when stomata are closed due to water limitation (Schreiber 2010). Root soil interfaces are characterized by the apoplastic deposition of suberin (Schreiber et al. 1999). Best known is the endodermis forming a root internal apoplastic barrier separating the central cylinder of the primary root from the cortex. However, the actual interface of roots directly facing the soil environment is formed by the rhizodermis and the hypodermis. The primary cell walls of these cell layers, especially the single or multi-layered hypodermis, are also characterized by the deposition of suberin (Hose et al. 2001; Serra et al. 2022). Tubers as storage organs of plants are characterized by a multi-layered periderm, which is suberized (Lulai and Corsini 1998). Finally, roots and shoots in their secondary developmental stages with a radial growth of thickness develop multi-layered suberized tissues as plant/environment interfaces.

Both, cutin and suberin are composed of oxygenated fatty acids, with varying chain lengths, which are polymerized (Kolattukudy 1980). Major cutin and suberin monomers are ω-hydroxy fatty acids and *α*,*ω*-diacids. The cutin polymer is always modified by the deposition of cuticular wax (e.g., linear, long-chain fatty acids, alcohols, aldehydes, alkanes, and esters) sealing the polymer and thus rendering it highly impermeable for water and dissolved substances (Kunst and Samuels 2003). The suberin polymer may contain wax molecules (Schreiber et al. 2005) or not (Schreiber et al. 1999; Teixeira and Pereira 2010). Cutin, suberin, and wax biosynthesis have intensively been investigated in the last 2 decades and many genes and enzymes involved have successfully been identified and characterized (Nawrath 2002; Samuels et al. 2008; Vishwanath et al. 2015; Fich et al. 2016).

Whereas there is no doubt that plant cuticles are highly efficient and impermeable polymer membranes, perfectly protecting plants from uncontrolled desiccation (Riederer and Schreiber 2001; Yeats and Rose 2013), this must not necessarily be the case with suberized cell walls. With the suberized periderm of potato, it was shown that the native periderm already formed a very efficient transpiration barrier directly after digging out from soil (Schreiber et al. 2005). Upon storage up to 4 weeks in the dark at ambient temperature and humidity, even a strong induction of suberin and wax biosynthesis was induced. The final water permeability of native potato periderm was further decreased by a factor of ten and was not different from leaf cuticles. However, water permeability of the wound periderm of potato, although it contained comparable amounts of suberin and wax, failed to form a transpiration barrier, since its water permeability was 100–1000 times higher compared to the native periderm.

Based on these contradictory results obtained with potatoes in the past, we investigated a series of further suberized tissues isolated from different plant organs (roots, tubers, and stems) from 5 different species in order to cover a broad spectrum of suberized tissue investigating their barrier properties. The anatomy (number of suberized cell layers) and the chemical composition (suberin and wax) of the various suberized samples was characterized and related to its properties as a water barrier, quantified by measuring transpiration kinetics. This larger set of data on the structure and function of suberized plant cell walls should help to estimate to what extent suberized plant/environment interfaces form transpiration barriers as efficient as plant cuticles or not.

## Materials and methods

### Selection and preparation of suberized tissues

Roots of *Clivia miniata* (Lindl.) Regel and *Monstera deliciosa* Liebm. were harvested from plants growing in the IZMB in Bonn. Soil-grown roots of *C. miniata* were carefully washed to remove adhering soil particles, whereas the air-exposed roots, which were slightly green due to photosynthetic pigments, were collected from the surface of the soil. Aerial roots of *M. deliciosa* were separated into young aerial roots (root tips with a smooth surface) and mature aerial roots (with a rough surface). Tubers of *Solanum tuberosum* L. var. WEGA were purchased from a local supermarket. Tubers of *Manihot esculenta* Crantz cultivated in a greenhouse at IGB-2 at Forschungszentrum Jülich GmbH (Jülich, Germany) were used for periderm isolation directly after harvest (fresh tubers) and after 3-week storage (stored tubers) at ambient temperature and humidity in the dark. Bark from *Malus domestica* Borkh. cv. Pinova was collected from 21-year-old trees growing in an orchard of the Institute of Horticultural Production Systems at the Hannover University.

The cylindrical roots of *C. miniata* and *M. deliciosa* were cut into 1.5–2 cm sections and the diameter was measured using a vernier caliper. Disks were punched out from *M. esculenta* and *S. tuberosum* skins with a cork borer (1.0 cm diameter) carefully avoiding any regions with lenticels. Bark samples from *M. domestica* were cut into sections of 1 cm^2^ with a razor blade. Suberized tissues from all samples were enzymatically isolated using 2% cellulase (Novozymes) and 2% pectinase (Novozymes) dissolved 0.01 M citric buffer (Carl Roth) with the pH adjusted to 3.0 (Vogt et al. 1983; Schönherr and Riederer 1986). The enzyme solution contained 1 mM of NaN_3_ (Fluka) to prevent microbial growth. The solution was changed once in 2 days until all suberized tissues were free from cellular debris. Isolated tissues were washed first with 0.01 M borate buffer (Carl Roth), adjusted to pH 9.0, and finally washed with deionized water. The cylindrical tissues of roots were carefully cut over the length in longitudinal sections and were fixed using paper clips on Teflon strips to carefully flatten them. A gentle air stream was used to air-dry isolated suberized tissues, which were stored in Petri dishes for 2–3 months at room temperature until the experiments were performed. In addition to the suberized samples, transpiration measurements were conducted with open transpiration chambers representing no barrier at all. Measurements with pure cellulose filter mimicking a primary carbohydrate cell wall without any further lipophilic modification were performed as well.

### Fluorescence microscopy

Freehand cross-sections were made for *C. miniata* and *M. deliciosa* roots with a razor blade. For other species, fresh samples were cut to a size of 1 cm × 0.2 cm (length × width) and semi-thin sections of about 20 µm thickness were made using a cryostat microtome (Microm HM 500 M, Microm International, Walldorf, Germany). Suberized cell walls were stained with 0.01% (w/v) Fluorol Yellow 088 (Sigma Aldrich) for 1 h and samples were rinsed with water before microscopic investigation (Brundrett et al. 1991). Cross-sections were analyzed by fluorescence microscopy using an ultraviolet (UV) filter set (excitation filter BP 365, dichroic mirror FT 395, barrier filter LP 397; Zeiss). Images were made with a Canon EOS 600D camera at ISO 100–400.

### Chemical analysis of wax and suberin composition of suberized tissues

Wax analysis of the suberized tissues was performed as described in Baales et al. (2021). Wax was extracted by immersing isolated suberized tissues in chloroform (5 ml) overnight in glass vials kept on a rolling bench. Before extraction, chloroform was spiked with 20 µg tetracosane (100 µl of a solution of 10 mg tetracosane in 50 ml chloroform; Fluka) as an internal standard for wax quantification. The total solvent volume of wax extracts was reduced under a gentle stream of nitrogen gas to a final volume of 200 µl. For suberin analysis, wax-extracted samples were depolymerized using boron trifluoride/methanol (BF_3_/MeOH, Fluka) as described by Baales et al. (2021). Before extraction of the released monomers with chloroform, transesterified samples were spiked with 20 µg of dotriacontane (100 µl of a solution of 10 mg dotriacontane in 50 ml chloroform; Fluka) as internal standard. The final chloroform volume was reduced to 200 µl using a gentle stream of nitrogen.

Both wax and suberin samples were derivatized for 45 min at 70 °C using 20 µl each of pyridine (Sigma Aldrich) and BSTFA (N, N-bis-trimethylsilyl-tri-fluoroacetamide, Machery-Nagel). This converts free functional groups of alcohols and acids to trimethylsilyl-ethers and -esters. Wax and suberin samples (1 µl) were quantified by GC-FID (CG-Hewlett Packard 5890 series H, Agilent) analysis and individual wax and suberin compounds were identified by GC–MS (quadrupole mass selective detector HP 5971, Hewlett Packard, Agilent) analysis. 1 µl of the wax samples were on-column injected, whereas analysis of suberin samples was done using split/splitless injection. Identification of the compounds was done using a homemade MS library.

### Transpiration measurements of suberized tissues

Transpiration was measured by gravimetry (Schönherr and Lendzian 1981). Prior to the measurement, dry and brittle suberized tissues were equilibrated overnight in an atmosphere with 100% humidity, making them soft and flexible, which allowed to handle them carefully without the danger of breaking. Suberized samples were carefully mounted on water-filled stainless-steel transpiration chambers with an open circular area of 0.28 × 10^–4^ m^2^ across which transpiration was possible. Before starting the transpiration experiment a 10 µl drop of ethanol was applied to the outer surface. This allows detecting micro-defects invisible to the eye since suberized samples immediately turn dark with ethanol penetrating defect suberized tissues. The chambers were closed with a lid (inner opening 0.28 × 10^–4^ m^2^).

Transpiration chambers were placed upside-down in an air-tight polyethylene box containing freshly activated silica at 25 °C, resulting in 2% humidity. Water loss across the suberized tissues was measured every hour up to 6 h using an analytical balance (Sartorius) with a resolution of 0.1 mg. As references, the transpiration of water from an open chamber (upright chamber) and across a pure cellulose filter (thickness: 140 µm) mounted to transpiration chambers were measured. The amount of water lost from individual suberized tissues or control samples were plotted as a function of time, and the slopes of the linear regression lines (in g s^−1^) fitted to the transport kinetics were used to calculate permeances P (m s^−1^) using the formula: *P* = slope/A × ΔC (Schreiber and Schönherr 2009), where A (m^2^) corresponds to the exposed area across which transpiration took place (0.28 × 10^–4^ m^2^) and ΔC (g m^−3^) represents the driving force for the transpiration given by the density of water (10^6^ g m^−3^).

### Statistical analysis

The number of suberized cell layers in the different samples was determined with 6–10 representative microscopic pictures for each species (Fig. 2). Wax and suberin analyses were done using 3 replicates (Figs. 3, 4, 5, 6). The transpiration kinetics were measured with 5–10 isolated samples before and after wax extraction (Fig. 7). Results are given as means with standard deviations or box plots. *t*-Tests were conducted and the levels of significance are indicated in the figures as 95% (**) or 99% (***), respectively.

## Results

### Microscopic investigation of suberized tissues

**Fig. 1:**
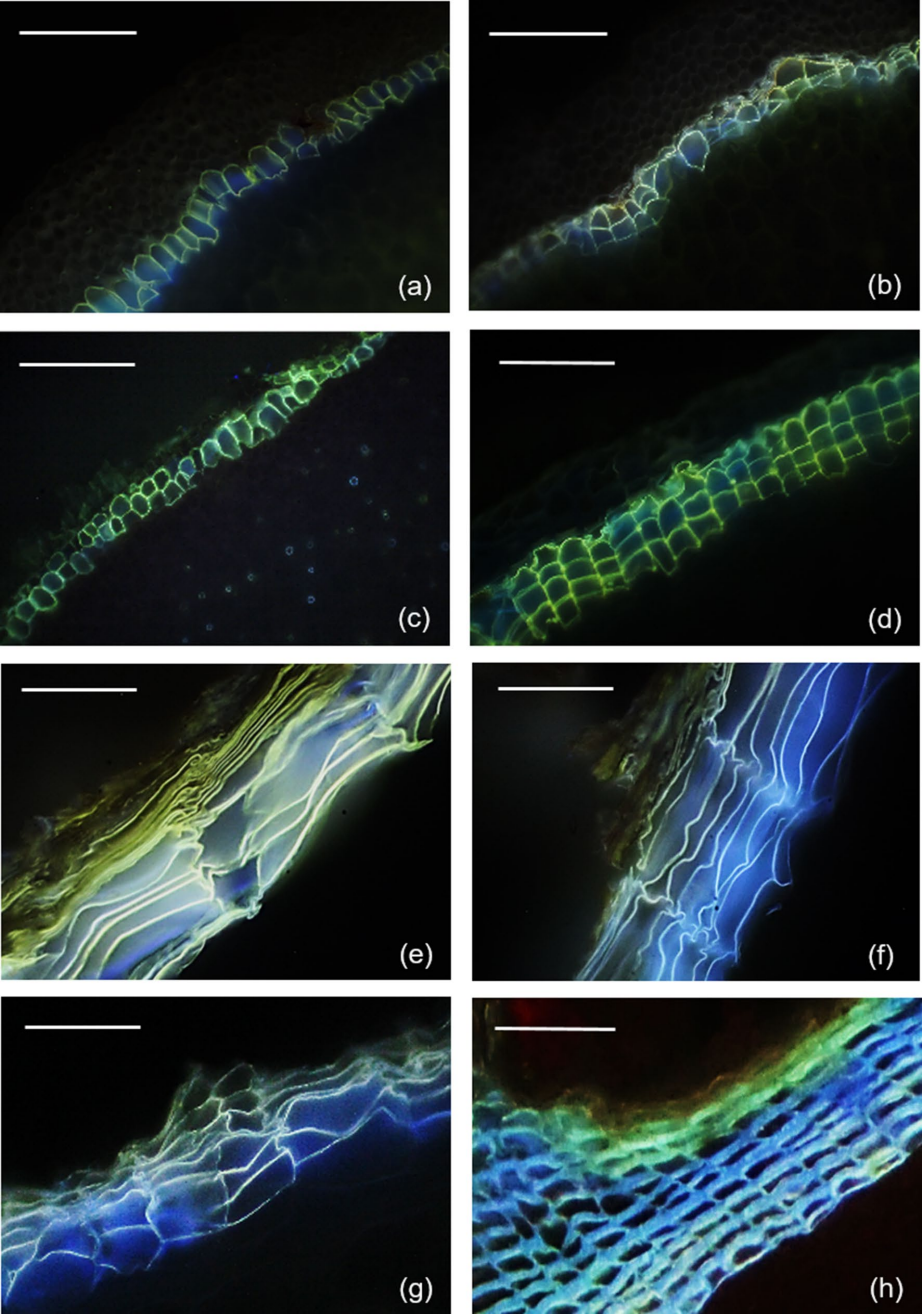
Fluorescence microscopic cross-sections of suberized tissues stained with Fluorol Yellow 088 and viewed at 365 nm. The presence of suberin is indicated by the greenish-yellow fluorescence. Suberized hypodermis isolated from **a** soil-grown roots of clivia (*Clivia miniata*) and from **b** air-exposed roots. Suberized hypodermis isolated from **c** young aerial roots of monstera (*Monstera deliciosa*) and from **d** mature aerial roots. Suberized periderm isolated from **e** freshly harvested cassava (*Manihot esculenta*) tubers, from **f** stored cassava tubers, and from **g** potato (*Solanum tuberosum*) tubers. Suberized shoot periderm isolated from **h** apple (*Malus domestica* cv. Pinova) bark. Bars = 100 µm

**Fig. 2:**
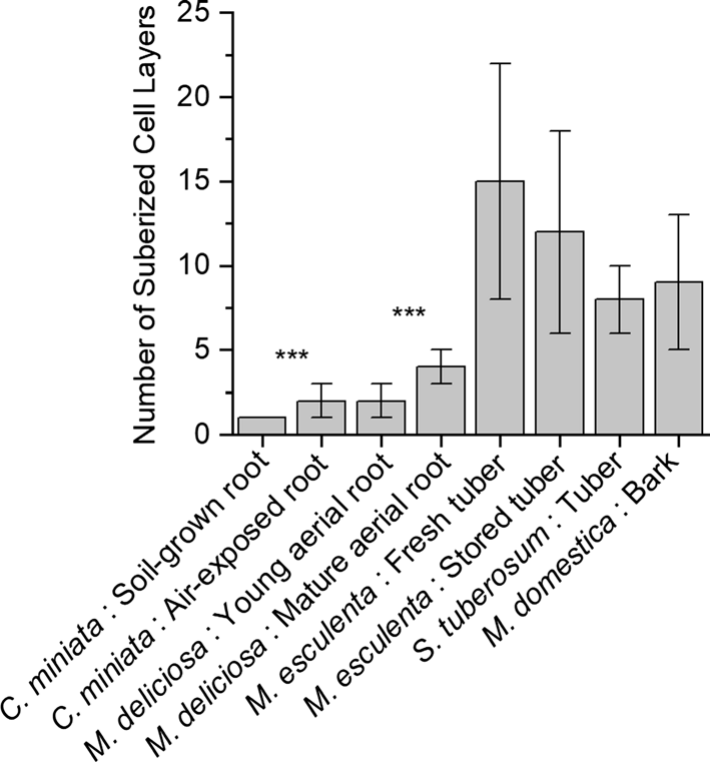
Number of cell layers in the different suberized tissues isolated from five different plant species (*Clivia miniata*, *Monstera deliciosa*, *Solanum tuberosum*, *Manihot esculenta,* and *Malus domestica*). The average number of cell layers given as means ± standard deviations was obtained by investigating at least 5 or more independent microscopic cross-sections of each sample. Asterisks indicate a significant difference between the number of cell layers of soil-grown and air-grown *Clivia* roots and of young and mature aerial root of *Monstera*, respectively (*** = 99%)

Cross-sections of the isolated suberized tissues were observed using fluorescence microscopy (Fig. 1a–h). Suberized cell walls appeared greenish/yellow or sometimes bluish/yellow after Fluorol Yellow 088 staining under UV light (395 nm). In soil-grown roots of *C. miniata* and air-exposed roots, the number of outer suberized cell layers varied between one and three (Figs. 1a, b, 2). Outer suberized tissues of young and mature aerial roots of *M. deliciosa* had two to four suberized cell layers (Figs. 1c, d, 2). Outer suberized periderms of tubers of *M. esculenta* had between 15 and 12 cell layers (Figs. 1e, f, 2). Suberized periderms from freshly harvested tubers (Fig. 1e) had several highly compressed suberized cells on the outer surface above the lately formed young suberized cells. In suberized periderms isolated from *M. esculenta* after storage for 3 weeks, these highly compressed layers were not intact anymore and to some extent thus missing (Fig. 1f). Periderms of *S. tuberosum* had 7 to 9 cell layers (Figs. 1g, 2). Suberized bark isolated from *M. domestica* shoots had between 8 and 10 suberized cell layers often only faintly stained (Figs. 1h, 2).

### Amounts and composition of wax extracted from suberized tissues

**Fig. 3:**
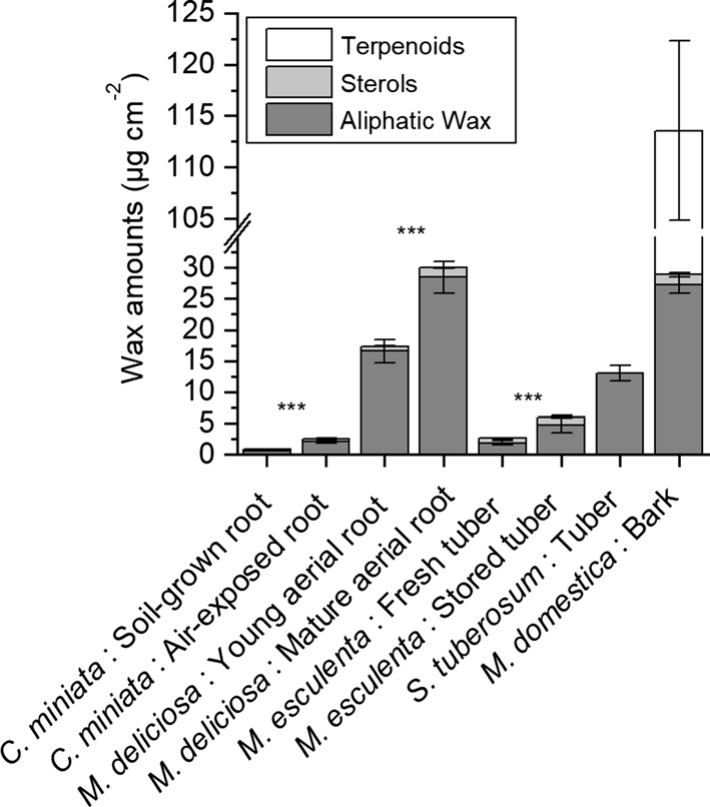
Total amounts (µg cm^−2^) of soluble wax extracted with chloroform from the different suberized tissues isolated from five different plant species (*Clivia miniata*, *Monstera deliciosa*, *Solanum tuberosum*, *Manihot esculenta,* and *Malus domestica*). Waxes are classified into 3 groups: aliphatic wax (linear long-chain aliphatic wax molecules), sterols (cyclic sterols), and terpenoids (triterpenoids and sesquiterpenoids). Data points represent means with standard deviations (*n* = 3). Asterisks indicate a significant difference between aliphatic wax amounts of soil-grown and air-exposed *Clivia* roots, of young and mature aerial root of *Monstera* and of fresh and stored Cassava tubers, respectively (*** = 99%)

**Fig. 4:**
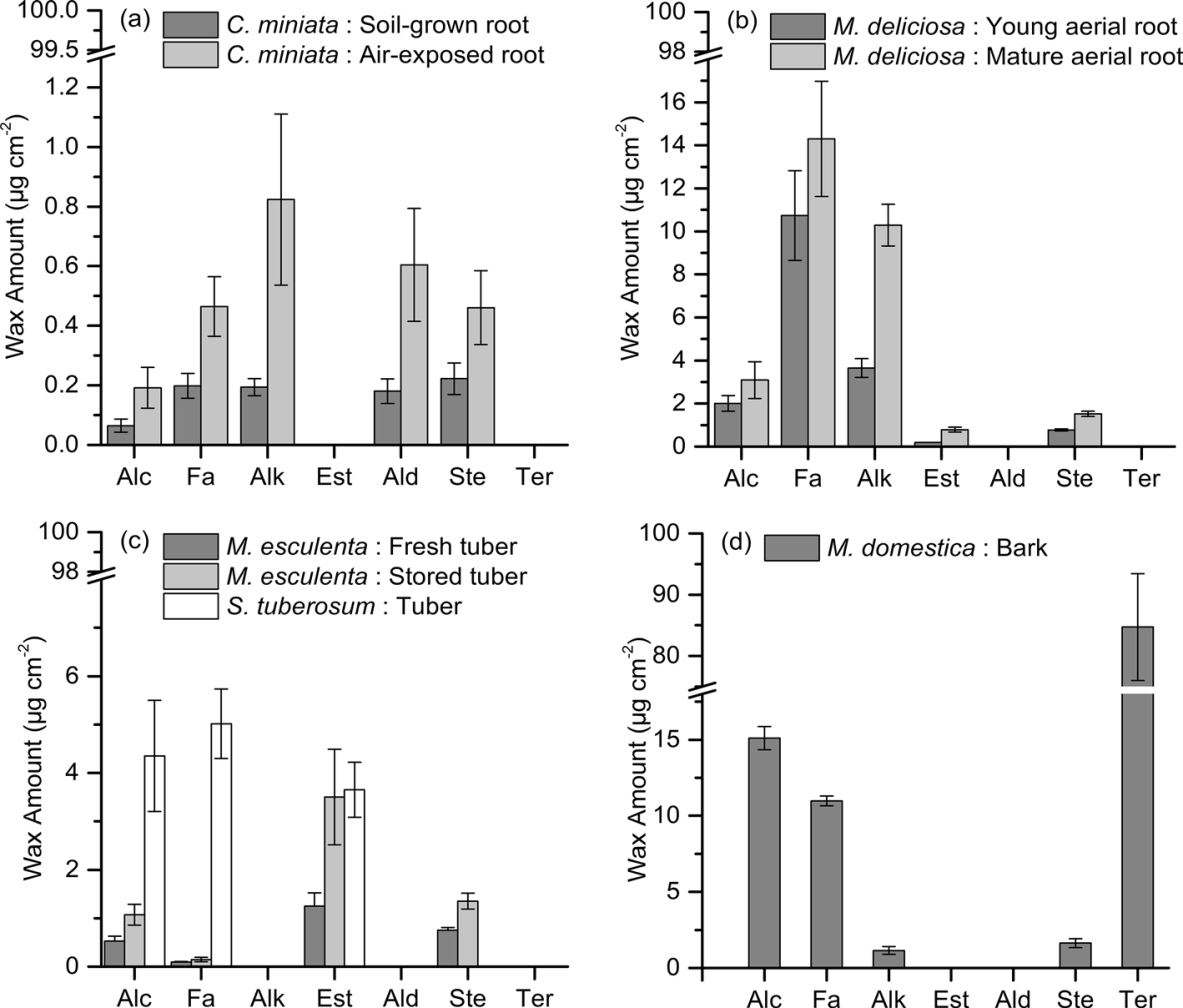
Substance classes of soluble waxes (µg cm^−2^) extracted with chloroform from the different suberized tissues isolated from five different plant species (*Clivia miniata*, *Monstera deliciosa*, *Solanum tuberosum*, *Manihot esculenta,* and *Malus domestica*). Besides sterols and terpenoids, amounts of aliphatic waxes are separated into alcohols (Alc), fatty acids (Fa), alkanes (Alk), esters (Est), aldehydes (Ald), sterols (Ste) and, terpenoids (Ter). **a** Wax composition of the suberized hypodermis isolated from soil-grown roots and air-exposed roots of *Clivia miniata*. **b** Wax composition of the suberized hypodermis isolated from young aerial roots and mature aerial of *Monstera deliciosa*. **c** Wax composition of the suberized periderms isolated from freshly harvested tubers and 3 weeks stored tubers *Manihot esculenta* and from *Solanum tuberosum*. **d** Wax composition of the suberized bark isolated from shoots of *Malus domestica*. Data points represent means with standard deviations (*n* = 3)

Wax amounts extracted from suberized tissues varied among different species both in amount and composition (Fig. 3). The detected wax monomers are separated into aliphatic wax amounts (linear, long-chain aliphatic wax monomers), sterols, and terpenoids. Sterols (stigmasterol and *β*-sitosterol) were detected only in minor and comparable amounts in nearly all samples except for potato. Fairly high amounts (84.7 ± 8.7 µg cm^−2^) of terpenoids were detected only in bark samples isolated from *M. domestica* (Figs. 3 and 4d). The highest amount of aliphatic waxes was found in suberized tissues isolated from mature aerial roots of *M. deliciosa* (28.5 ± 2.5 µg cm^−2^) and suberized bark of *M. domestica* (27.2 ± 0.7 µg cm^−2^), respectively (Fig. 3). In suberized tissues isolated from soil-grown *C. miniata* roots, aliphatic wax amounts were only 0.9 ± 0.1 µg cm^−2^, whereas higher wax amounts of 2.5 ± 0.2 µg cm^−2^ were detected in air-exposed roots (Fig. 3). The average amount of total wax in suberized tissue of young aerial roots of *M. deliciosa* was 17.4 ± 1.9 µg cm^−2^ (Fig. 3). Freshly isolated periderms of *M. esculenta* had only 2.6 ± 0.4 µg cm^−2^ aliphatic wax and amounts increased after 3 weeks storage to 6.0 ± 1.3 µg cm^−2^ (Fig. 3). The amounts of wax extracted from *S. tuberosum* were 13.0 ± 1.3 µg cm^−2^ (Fig. 3).

The fraction of the aliphatic wax molecules was composed of the substance classes alcohols, acids, alkanes, esters, and aldehydes (Fig. 4) of varying chain lengths (C_16_–C_34_). In *C. miniata*, the substance classes alcohols, acids, alkanes, and aldehydes were detected and amounts of all substance classes were significantly higher in air-exposed roots (Fig. 4a). In *M. deliciosa* alcohols, acids, alkanes, and esters were detected and amounts of all substance classes increased in mature aerial roots with the alkanes showing the highest increase (Fig. 4b). In *M. esculenta* and *S. tuberosum*, only three substance classes, namely alcohol, acids, and esters, were present and the amount of alcohols and esters increased two- to threefold in stored *M. esculenta* periderms (Fig. 4c). In *S. tuberosum*, the fraction of esters was essentially composed of ferulic acid esters of the chain lengths C_20_ to C_32_. In *M. domestica*, alcohols and acids were the dominating substance classes of wax whereas alkanes were only present in minor amounts (Fig. 4d).

### Amounts and composition of suberin monomers depolymerized from suberized tissues

**Fig. 5:**
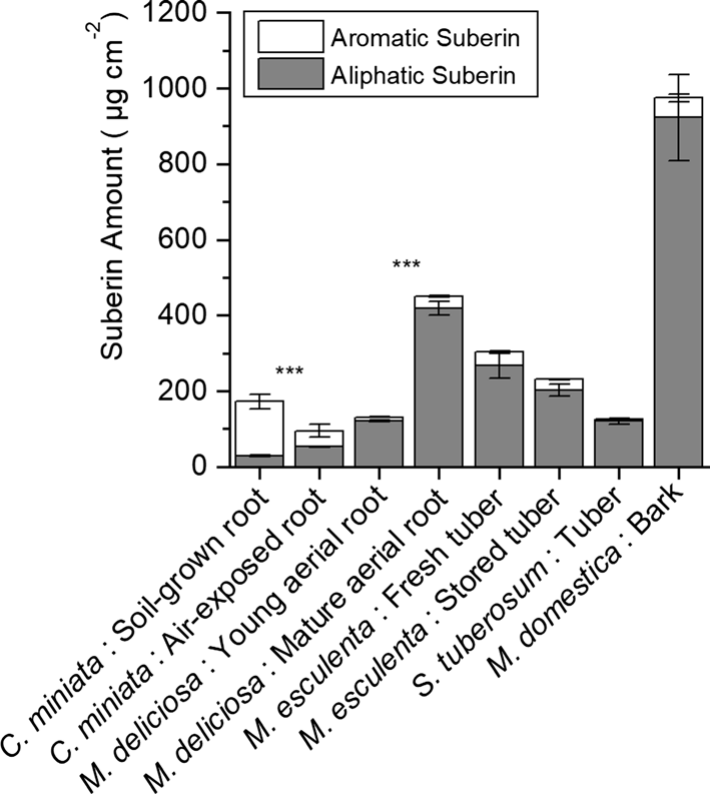
Total amounts (µg cm^−2^) of aromatic (ferulic and coumaric acids) and aliphatic suberin (linear long-chain aliphatic suberin monomers) obtained after depolymerization of the different wax-extracted suberized tissues isolated from five different plant species (*Clivia miniata*, *Monstera deliciosa*, *Solanum tuberosum*, *Manihot esculenta,* and *Malus domestica*). Data points represent means with standard deviations (*n* = 3). Asterisks indicate a significant difference between aliphatic suberin amounts of soil-grown and air-exposed *Clivia* roots, of young and mature aerial root of *Monstera* and of fresh and stored Cassava tubers, respectively (*** = 99%)

**Fig. 6:**
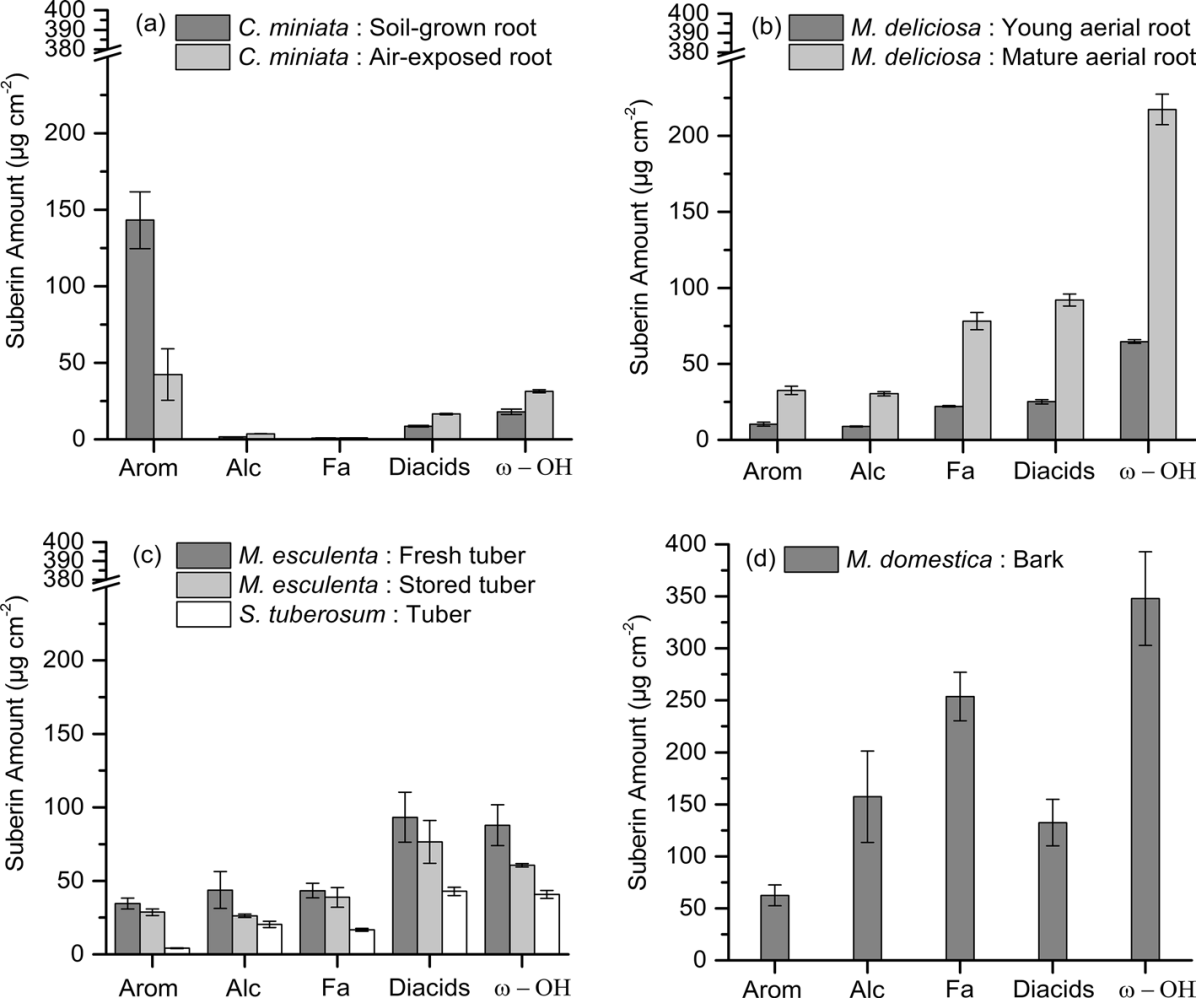
Amounts of the different substance classes of suberin monomers (µg cm^−2^) obtained after depolymerization of the different suberized tissues isolated from five different plant species (*Clivia miniata*, *Monstera deliciosa*, *Solanum tuberosum*, *Manihot esculenta,* and *Malus domestica*). Substance lasses are separated into aromatics (Arom), primary alcohols (Alc), fatty acids (Fa), *α*,*ω*-diacids, and *ω*-hydroxy acids (*ω*-OH). **a** Substance classes of the suberized hypodermis isolated from soil-grown roots and air-exposed roots of *Clivia miniata*. **b** Substance classes of the suberized hypodermis isolated from young aerial roots and mature aerial of *Monstera deliciosa*. **c** Substance classes of the suberized periderms isolated from freshly harvested tubers and 3-week-stored tubers *Manihot esculenta* and from *Solanum tuberosum*. **d** Substance classes of the suberized bark isolated from shoots of *Malus domestica*. Data points represent means with standard deviations (*n* = 3)

Suberin content of the different suberized tissues varied between 100 and 1000 µg cm^−2^ (Fig. 5). Monomers obtained after suberin depolymerization were classified into aliphatic (linear, long-chain oxygenated fatty acids) and aromatic suberin (essentially coumaric and ferulic acids). Except for soil-grown roots of *C. miniata*, the aromatic fraction of the suberin polymer in the other samples amounted only to a few percent of the total suberin amounts (Fig. 5). In *C. miniata* roots, the aliphatic suberin amount in hypodermis was about twofold higher in air-exposed roots (52.4 ± 1.5 µg cm^−2^) when compared to soil-grown roots (29.1 ± 2.4 µg cm^−2^) (Fig. 5). In *M. deliciosa*, the total aliphatic suberin content was 120.70 ± 1.7 µg cm^−2^ in young aerial roots and it increased to 417.9 ± 18.2 µg cm^−2^ (Fig. 5). Upon 3 weeks of storage, the amount of aliphatic suberin in *M. esculenta* decreased from 268.2 ± 40.8 to 202.1 ± 20 µg cm^−2^ (Fig. 5). In *S. tuberosum*, the total aliphatic suberin content was 120.7 ± 7.9 µg cm^−2^ and the highest aliphatic suberin content of 891.2 ± 109.4 µg cm^−2^ was measured with the periderm isolated from *M. domestica* bark (Fig. 5). Dominating substance classes of the aliphatic suberin monomers detected in all samples after depolymerization were *ω*-hydroxy acids and *α*,*ω*-diacids (Fig. 6). In addition, varying amounts of linear long-chain alcohols and fatty acids were also released by transesterification (Fig. 6). The chain length of the aliphatic suberin monomers ranged from C_16_ to C_30_ (data not shown). Substance classes of suberin tissues did not change when comparing soil-grown with air-exposed *C. miniata* roots (Fig. 6a), young with mature aerial *M. deliciosa* roots (Fig. 6b), and fresh with 3-week-stored *M. esculenta* tubers (Fig. 6c).

### Rates of water loss (transpiration) from suberized tissues

**Fig. 7:**
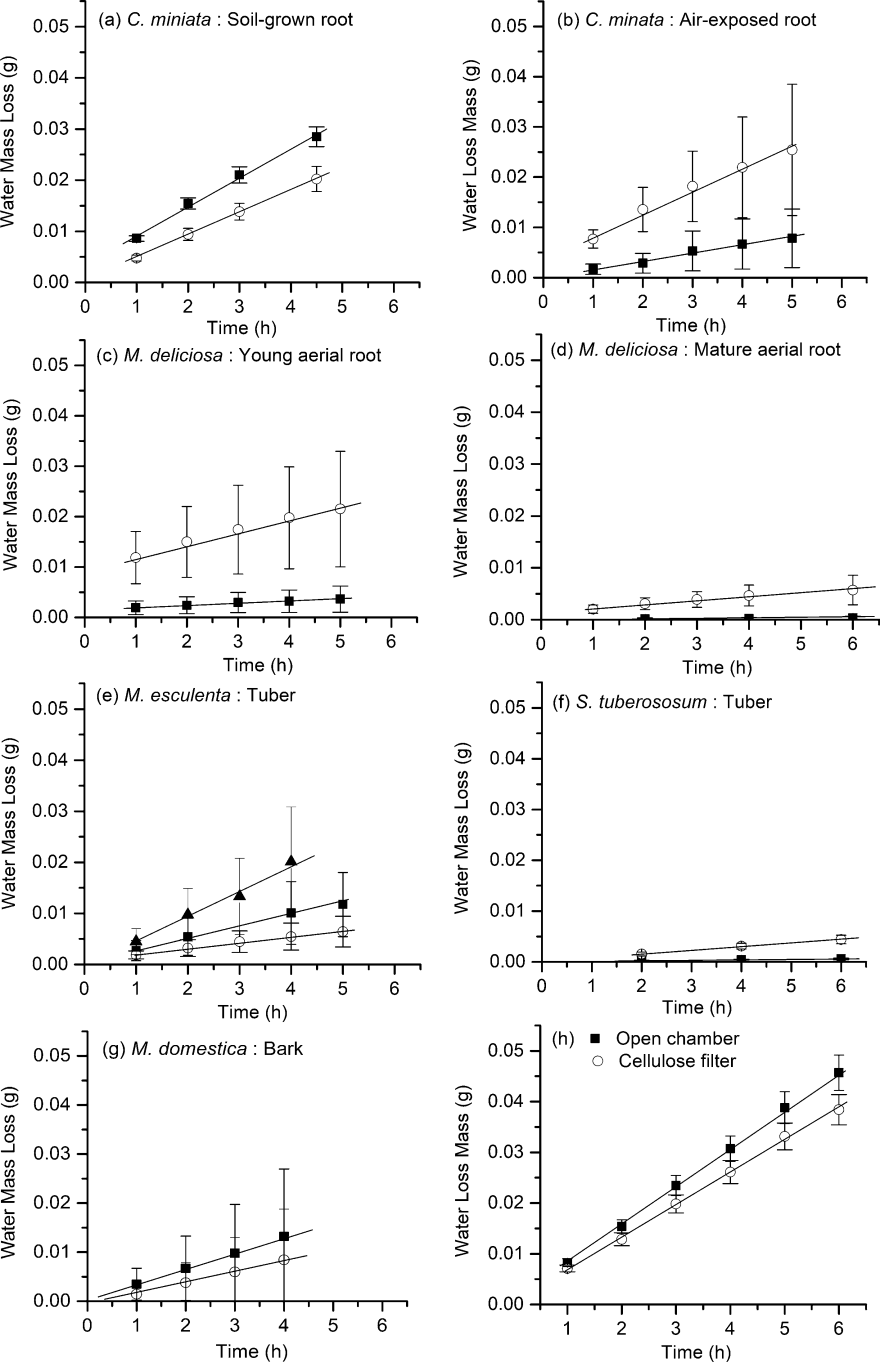
Transpiration kinetics (g h^−1^) measured for the different suberized tissues isolated from five different plant species (*Clivia miniata*, *Monstera deliciosa*, *Solanum tuberosum*, *Manihot esculenta,* and *Malus domestica*). **a–g** Intact suberized tissues (black squares) and wax-extracted suberized tissues (white circles) were compared. **e** Intact periderms isolated from 3-week-stored tubers of *Manihot esculenta* (black triangles) are shown in comparison to intact periderms isolated from freshly harvested tubers of *M. esculenta* (black squares). **h** Transpiration kinetics were measured with cellulose filters (white circles) and with open transpiration chambers (black square). Data points represent means with standard deviations of (*n* ≥ 10)

**Fig. 8:**
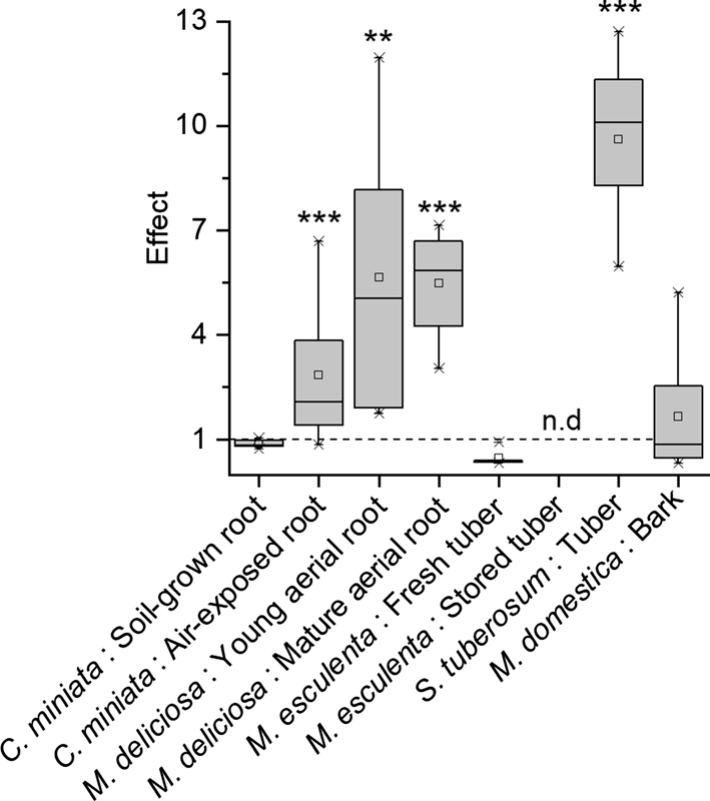
Effects of wax extraction on transpiration measured with suberized tissues isolated from five different plant species (*Clivia miniata*, *Monstera deliciosa*, *Solanum tuberosum*, *Manihot esculenta,* and *Malus domestica*). Effects were calculated by dividing the slopes of transpiration kinetics of wax-extracted suberized tissues by the slopes of transpiration kinetics measured with intact suberized tissues. As a reference (dotted line), the effect of 1 is shown, indicating that there was no change in transpiration after the extraction of wax from the isolated suberized tissue. Means (*n* ≥ 5) with standard deviations are shown. n.d. = not determined. Asterisks indicate a significant different effect from 1 (** = 95%; *** = 99%)

Linear transpiration kinetics were obtained plotting the amounts of water lost from the transpiration chambers vs. time (Fig. 7). The highest transpiration rates were measured with open chambers and cellulose filters (Fig. 7h) and slopes of the transpiration kinetics were not significantly different between the two samples. The lowest rates of transpiration were measured with periderms isolated from *S. tuberosum* (Fig. 7f). The transpiration kinetics of all other samples were between these upper and lower value ranges (Fig. 7b–e, g). Water permeability of the suberized samples increased by factors of 1.7 ± 1.7 (*M. domestica*), 2.8 ± 1.8 (the exposed root of *C. miniata*), 5.6 ± 3.8 (young roots of *M. deliciosa*), 9.2 ± 7.7 (mature roots of *M. deliciosa* and 9.6 ± 2.1 (*S. tuberosum*) after solvent extraction of waxes with chloroform (Fig. 8). With the suberized hypodermis isolated from *C. miniata* soil-grown roots and the periderm isolated from *M. esculenta* fresh tubers, the rates of water loss were slightly decreased after wax extraction (Fig. 8).

**Fig. 9:**
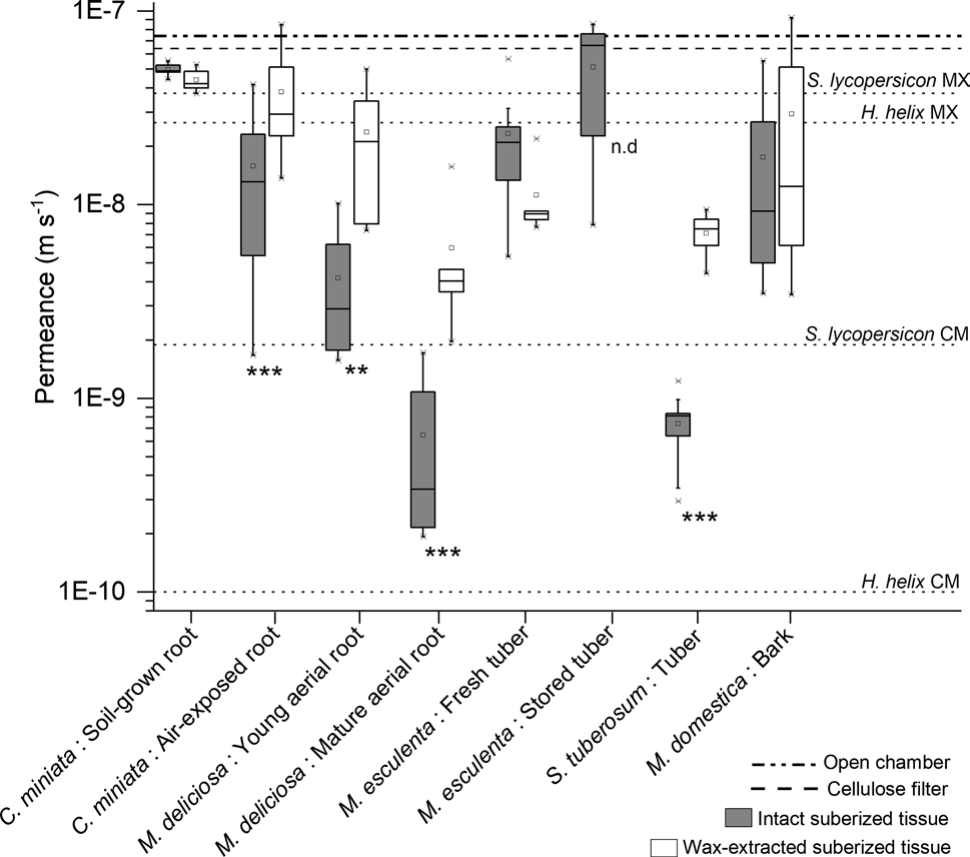
Box plots of permeances P (m s^−1^) calculated from the regression lines fitted to the transpiration kinetics measured with suberized tissues isolated from five different plant species (*Clivia miniata*, *Monstera deliciosa*, *Solanum tuberosum*, *Manihot esculenta*, and *Malus domestica*). Intact suberized tissues (grey box plots) and wax-extracted suberized tissues (white box plots) were compared. *P* (dash dot line) of the open transpiration chamber (7.4 × 10^–08^ m s^−1^) and *P* (dashed line) of the cellulose filter (6.4 × 10^–08^ m s^−1^) is given as “upper” reference lines. *P* (dotted line) of the leaf cuticle isolated from *Hedera helix* 9.9 × 10^–11^ m s^−1^ and fruit cuticle from *Solanum lycopersicon* 1.9 × 10^–9^ m s^−1^; Schönherr and Lendzian (1981) is given as a “lower” reference line. Box plots with medians (line in the box), means (square in the box), whiskers (10–90% of the values), and outliers (crosses) are given (*n* ≥ 5; n.d. = not determined. Asterisks indicate a significant difference between permeances of intact periderms and wax-extracted periderms (** = 95%; *** = 99%)

From the slopes of the regression lines fitted to transpiration kinetics, permeances P (m s^−1^) were calculated for suberized intact periderm, wax-extracted periderm, the open transpiration chamber, and the cellulose filter (Fig. 9). Permeances obtained for intact suberized samples varied between the lowest value of 6.5 × 10^–10^ (± 6.2 × 10^–10^) m s^−1^ measured with matured aerial roots of *M. deliciosa* and the highest value of 5.1 × 10^–08^ (± 3.0 × 10^–08^) m s^−1^ measured for stored tubers of *M. esculenta* (Fig. 9). Permeances obtained for wax-free suberized samples varied between the lowest value of 5.8 × 10^–09^ (± 5 × 10^–09^) m s^−1^ measured with matured aerial roots of *M. deliciosa* and the highest values of 4.4 × 10^–8^ (± 5.4 × 10^–08^) m s^−1^ measured for air-exposed roots of *C. miniata* (Fig. 9). Permeances obtained for the open chamber and the cellulose filter were 7.4 × 10^–08^ (± 5.6 × 10^–09^) m s^−1^ and 6.4 × 10^–08^ (± 5.0 × 10^–09^) m s^−1^ (Fig. 9).

## Discussion

Extremely steep gradients with water potentials of about – 160 MPa (about 30% relative humidity) in the atmosphere, strongly driving foliar transpiration, can be followed within hours by very flat gradients with water potentials higher than – 1.5 MPa (about 99–100% relative humidity), hardly causing any gradient for an efficient transpiration of water from the leaf (Milburn 1979; Pickard 1981; Chen et al. 1999). Due to these rapid changes in water vapor gradients between the inside (nearly 100% relative humidity) and the outside of leaves, throughout their life period leaves need constant and nearly perfect protection from uncontrolled water loss. There is no doubt, that this protection is successfully provided by the plant cuticle sealed with waxes (Schreiber and Schönherr 2009), which is highly impermeable for water, especially when compared to stomatal transpiration (Grünhofer et al. 2022). Permeances of plant cuticles, efficiently protecting leaves and fruits from uncontrolled water loss, cover a range between 10^–10^ and 10^–9^ m∙s^−1^ (Schreiber and Riederer 1996). As examples for representative permeances for cuticles, P of *Hedera helix* leaf cuticle and of *Solanum lycopersicon* fruit cuticle can be given here (Fig. 9), having a value of 0.9 × 10^–11^ m s^−1^ and 1.9 × 10^–09^ m s^−1^ respectively (Schönherr and Lendzian 1981). Upon wax extraction, permeances of wax-free cuticles of *Solanum lycopersicon* were 3.7 × 10^–08^ m s^−1^ and permeances of *Hedera helix* were 2.6 × 10^–08^ m s^−1^ (Schönherr and Lendzian 1981). Thus, wax extraction resulted in 20- (*Solanum lycopersicon*) and 265-fold (*Hedera helix*) increased permeances.

The highest possible transpiration rate of water, which can theoretically be measured with the system used here, is given by the permeance of an open transpiration chamber without any membrane mounted. This measurement resulted in a permeance of 7.4 × 10^–08^ m s^−1^ (Fig. 9). The fact that a primary carbohydrate cell wall, without any further aromatic (lignin) or aliphatic (suberin or cutin) modification, does not represent an efficient transpiration barrier is shown for the filter membrane made of pure cellulose, which was mounted to the transpiration chamber. Although the cellulose filter investigated here had a thickness of 140 µm, which is by far thicker than a regular primary carbohydrate cell wall of a leaf, varying about 1–2 µm (Moghaddam and Wilman 1998), the permeance was 6.4 × 10^–08^ (± 5 × 10^–09^) m s^−1^ which is statistically not different from the value measured for an open chamber (Fig. 9). It is remarkable that permeances of wax-free cuticles were only about two- to threefold lower than the permeance of the open transpiration chamber (Fig. 9). All further permeances measured here with the different suberized cell wall samples isolated from roots, tubers or shoots, are located between the low values of cuticles and the high values of cellulose filter and the open transpiration chamber (Fig. 9).

In comparison to the atmosphere surrounding the leaves, which is characterized by wide variation in water potentials reaching very low values, the range and the temporal variation in soil by far less pronounced. Field capacity is defined as the maximum amount of water absorbed by the soil, water potentials are very close to 0 ( – 0.03 MPa), whereas a potential of – 1.5 MPa is already defined as the permanent wilting point of plants (Kramer and Boyer 1995). At soil water potential of – 1.5 MPa, corresponding to a relative humidity of nearly 99% (Milburn 1979), most herbaceous plants and crops cannot take up water anymore and will start to wilt. This permanent wilting point of – 1.5 MPa leads to the conclusion that the main problem for plants, when dealing with water shortage, is not related to the fact that they do not have an efficient transport barrier on the root surface, protecting them from desiccation, but obviously plants are not able to decrease their internal water potentials to values significantly more negative than – 1.5 MPa. Consequently, at a soil water potential of – 1.5 MPa or lower, the driving force for the passive water diffusion usually directed inwards is inversed leading to wilting of plants already at 99% soil humidity.

Thus, it is not too surprising that the permeance measured with the suberized hypodermal cell layers, isolated from soil-grown roots of *C. miniata*, was 5 × 10^–08^ (± 3.2 × 10^–09^) m s^−1^, which is nearly as high as the cellulose membrane and the open transpiration chamber (Fig. 9). Upon wax extraction, permeance was statistically not significantly different from the intact hypodermis (Fig. 8), indicating that there is hardly any diffusional barrier for water developed with the outer suberized cells of soil-grown *C. miniata* roots. The suberized hypodermis isolated from *C. miniata* roots exposed to the atmosphere had fivefold lower permeance of 1.6 × 10^–08^ (± 1.2 × 10^–08^) m s^−1^ compared to the soil-grown roots. Upon wax extraction the permeance increased on an average threefold, indicating that the wax in the suberin polymer of the air-exposed roots established this, albeit very weak, diffusional barrier for water and not the slightly increased amounts of aliphatic suberin (Fig. 5).

Very different from *C. miniata*, the suberized hypodermis isolated from aerial roots of *M. deliciosa* had fairly low permeances between 4.2 × 10^–09^ (± 3 × 10^–09^) m s^−1^ measured for the young still developing aerial root tip and 6.5 × 10^–10^ (± 6.2 × 10^–10^) m s^−1^ measured for the mature aerial root zone (Fig. 9). These values nearly match permeances located in the upper range of isolated cuticular membranes (Schreiber and Schönherr 2009). Thus, aerial roots of *M. deliciosa*, facing the steep gradient in water potential between the roots and the atmosphere, obviously need an efficient transpiration barrier for survival. The intensity of suberization (Fig. 5), and the amounts of wax (Fig. 3), being significantly higher in *M. deliciosa* compared to *C. miniata*, established this pronounced transpiration barrier (Fig. 3), which is again largely lost upon wax extraction (Fig. 8). This emphasizes the significance of the wax in establishing a transpiration barrier, as it is also the case with leaf cuticles. In addition, it is worth pointing out that the number of suberized cell layers is on average only twofold higher with *M. deliciosa* (2–4 cell layers) compared to *C. miniata* (1–2 cell layers), whereas the permeance of the suberized tissue of *M. deliciosa* compared to *C. miniata* is on average 1–2 orders of magnitude lower (Fig. 9). Thus, it is not so much an increase in the number of suberized cell layers and in suberin amounts reducing water permeability, but in wax deposition establishing the transpiration barrier of aerial roots of *M. deliciosa*.

Tuber and storage roots as subterranean storage organs of plants are growing all their life span in soil and they develop fairly thick outer periderms as interfaces towards the soil environment. This is also the case here with *M. esculenta*, characterized by 12 to 15 suberized cell layers, and with *S. tuberosum* having a slightly lower number of about 10 suberized cell layers (Fig. 1 and 2). Therefore, it is very surprising that the periderm of *M. esculenta* nearly completely failed to establish reasonable transpiration barriers, whereas it was exactly the opposite with the periderm of *S. tuberosum*, forming a highly efficient transpiration barrier (Fig. 9). Permeances measured with the periderms of *M. esculenta* varied between 2.3 × 10^–08^ m s^−1^ and 5.1 × 10^–08^ m s^−1^, which is comparable to the values obtained with soil-grown *C. miniata* roots and already very close to the values obtained with the cellulose filter and the open transpiration chamber (Fig. 9). However, permeances obtained for *S. tuberosum* were 7.4 × 10^–10^ (± 2.7 × 10^–10^) m s^−1^, which is 1 to 2 orders of magnitude lower compared to the permeances of *M. esculenta*. The permeances measured here for *S. tuberosum* also fit values published for *S. tuberosum* in the past (Schreiber et al. 2005) and they are in the range of permeances (10^–11^–10^–09^ m s^−1^) published for highly water-impermeable cuticular membranes (Schreiber and Riederer 1996).

Different from *S. tuberosum*, where it was shown that upon 4-week-storage permeances of periderms decreased by 1 order of magnitude (Schreiber et al. 2005), permeances of *M. esculenta* periderms isolated after 3 weeks of storage were statistically not different from freshly isolated periderms (Fig. 9), although aliphatic wax amounts of *M. esculenta* periderms increased by about twofold during 3-week storage (Fig. 3). This completely opposing behavior between the periderm of *S. tuberosum*, forming a very efficient transpiration barrier, and the periderm of *M. esculenta*, completely lacking the ability to form an efficient water barrier (Fig. 9), protecting tubers against water loss, fits the well-known difference in shelf-life between both tubers. Whereas *S. tuberosum* tubers ideally can be stored for several months (Alamar et al. 2017), *M. esculenta* tubers rapidly start to deteriorate within 24 h after harvest (Saravanan et al. 2016). Besides many other physiological and enzymatic processes leading to rapid deterioration and loss of nutritional quality of harvested *M. esculenta* tubers, this total failure of the periderm protecting the tubers from rapid dehydration represents another significant factor for the pronounced postharvest losses of *M. esculenta*. Covering the tubers with paraffin wax, which will reduce tuber dehydration, can delay the postharvest deterioration by a couple of weeks (Uchechukwu-Agua et al. 2015). At the moment, it remains an interesting and unsolved question, why *M. esculenta* completely fails to establish an efficient transpiration barrier protecting the tubers, whereas *S. tuberosum* is highly successful?

One could speculate that this difference between *M. esculenta* and *S. tuberosum* establishing a transpiration barrier could be related to additional yet unknown differences in the polyphenolic cell wall modifications of both periderms, which, however, would need to be investigated in the future. Another reasonable explanations could be the completely different ontogenetic origin of both types of tubers. The tuber of *M. esculenta* develops from the root, whereas the tuber of *S. tuberosum* originates from a shoot growing horizontally belowground. Potentially the genetic and biochemical machinery, leading to a pronounced suberin and wax biosynthesis, is activated a lot more in a tuber being homologous to a plant shoot, naturally facing the atmosphere, instead of a tuber originating from a root, normally facing the soil environment. A further explanation could be the functions of the tuber of *S. tuberosum* and storage root of *M. esculenta*. Potato tubers allow re-growth after dormancy protecting the apical and lateral axillary buds and the resources for re-growth from abiotic and biotic conditions (Suttle 2004), while the storage roots of *M. esculenta* do not experience times of dormancy but facilitate growth of the perennial shrub serving as a carbon sink and source tissue for growth (El-Sharkawy 2004). *Solanum* species show a huge variation in dormancy, and it is affected by pre- and postharvest environmental conditions (Sonnewald 2001; Suttle 2004). For example, the Chilean and European potatoes are believed to derived from a domestication event that took place in Peru at an altitude of 3000–4000 m. As a consequence, tubers of potatoes would need suberin barriers to withstand the abiotic and biotic conditions guaranteeing re-growth after the dormancy period in such an altitude. These potatoes migrated through hybridization with other Andean wild species to coastal Chile over time, which allowed the adaptation to temperate climates (reviewed by Ramsay and Bryan 2011). However, cultivars of the *S. tuberosum* L. Phureja Group occurred at the Eastern slope of the Andes from western Venezuela to central Bolivia in an altitude of 2000 to 3400 m (Ochoa 1990), and some cultivars lack tuber dormancy in this group (Ghislain et al. 2006). Thus, the dormancy and subsequently suberization of the tuber could be due to the environmental origins of the Chilean and European potato or due to a selection and breeding process as an adaptation to the Chilean coastal regions and subsequently to temperate climates. Such an adaptation or adaptation/breeding of cassava never occurred as the crop is only grown in the tropical region around the globe (reviewed by McKey and Delêtre 2017). Therefore, the discrepancy in periderm function between *M. esculenta* and *S. tuberosum* remains an interesting scientific as well as important applied research question to be analyzed in the future.

The last sample of suberized tissues analyzed here was the periderm isolated from *M. domestica* shoots. Although this periderm was characterized by the highest amount of wax molecules (Fig. 3) and suberin monomers (Fig. 5) of all samples investigated here, rates of water loss were surprisingly high (Fig. 7h). The permeance was about 1.1 × 10^–08^ (± 3.4 × 10^–08^) m s^−1^ and there was no significant increase in permeance after the extraction of wax 3 × 10^–08^ (± 1.8 × 10^–08^) m s^−1^ (Fig. 8). At the moment, we do not have a straightforward explanation as, to why the periderm isolated from *M. domestica* shoots did not represent a reasonable transpiration barrier. Maybe different possibilities must be considered. First, periderms were isolated from still growing and therefore continuously radially expanding shoots, which could be a reason for the failure to establish an efficient transpiration barrier. In addition, compared to the other suberized samples, handling of the periderms isolated from *M. domestica* shoots and mounting to the transpiration chambers was fairly difficult, since shoot periderms were very brittle. It cannot be excluded that this caused some defects or cracks in the investigated periderms, which were not detectable and visible. Therefore, it is worth investigating the water permeability of periderms isolated from shoots in more detail in the future.

## Conclusion

From the data presented and discussed here, it can be concluded that there is no straightforward explanation why certain suberized tissue can form efficient transpiration barriers and others fail. For the soil-grown roots and considering the weak gradients for potential water loss it is understandable that an effective water barrier is not needed, whereas for aerial roots facing the atmosphere an efficient transpiration barrier was established. From a physiological point of view, it can be hypothesized that periderms of tubers, which are acting as storage organs of plants, should have a good transpiration barrier. This was the case for *S. tuberosum* but not at all for *M. esculenta*. This discrepancy remains unclear. Our data also clearly shows that the pronounced variations in wax and suberin amounts and composition do not lead to an easy explanation of why certain suberized tissue represents efficient transpiration barriers and others not. However, for those suberized tissues forming good transpiration barriers, it is evident that wax is essential for barrier formation, since upon wax extraction barrier properties are largely lost. Thus, biotechnological approaches trying to improve the transpiration barriers of suberized tissues should focus on the enhancement of wax biosynthesis.

### Author contribution statement

LS and TW obtained the grants to support this study. KS, VZ, TW, and LS designed the study and planned the experiments. KS and VZ conducted the experiments. All the authors analyzed the data. KS, VZ, TW, and LS wrote the manuscript. All the authors read and approved the manuscript.

## Data Availability

All data generated or analyzed during this study are included in this published article.
